# Empowerment, Participation, and Person‐Centeredness as Prerequisites for Health‐Promoting Settings in Everyday Practice: A Swedish Case Study

**DOI:** 10.1111/hex.70632

**Published:** 2026-03-01

**Authors:** Petra Nilsson Lindström, Sophie Schön Persson, Johanna Sjöbeck, Matilda Ahl, Kerstin Nilsson, Åsa Bringsén

**Affiliations:** ^1^ Faculty of Health Science Kristianstad University Kristianstad Sweden; ^2^ Skåne University Hospital Lund Sweden

**Keywords:** co‐creation, empowerment, health promotion, participation, person‐centeredness, qualitative collective case study, settings‐based approach

## Abstract

**Introduction:**

Health promotion in everyday settings is of great importance for both individual health and organizational sustainability. A settings‐based approach emphasizes how social, physical, and organizational contexts shape the conditions for health. Central principles in health promotion are participation, empowerment, and person‐centeredness, which emphasize the active role of the person or group in relation to the context. Although these principles are often highlighted in policy and theory, there is limited knowledge about how they are concretely translated into everyday practice within different contexts. The purpose of the study was therefore to explore how health promotion is conducted in different everyday settings, with a particular focus on how participation, empowerment, and person‐centeredness take shape in practice.

**Method:**

The study was conducted as an exploratory qualitative collective case study supported by the Storytelling dialog method. Four different health‐promoting settings in southern Sweden were studied: a preschool, a sports profile within an upper secondary school, a daily activity for persons with neuropsychiatric functionality disabilities, and municipal community development for seniors. Data collection consisted of participant observations and semi‐structured interviews, and the material was written into four case stories.

**Findings:**

The findings showed two interrelated themes: Practice‐embedded prerequisites for a setting approach and an experiential and behavioral process supporting health development in settings. The first theme describes how societal support, organizational structures, staff competence, and everyday interactions create supportive conditions for health promotion. The second theme illustrates how experiences of inclusion, belonging, and motivation emerge within these conditions and foster active engagement, agency, and ongoing health development in everyday settings.

**Conclusion:**

Participation, empowerment, and person‐centeredness appear to be mutually supporting and central to sustainable health promotion, regardless of setting. This study thus contributes to a deeper understanding of how these principles (participation, empowerment, and person‐centeredness) can be operationalized in everyday settings through a systems and process perspective illuminated from different contexts.

**Patient or Public Contribution:**

During data collection, representatives from each organization studied were either observed and/or given the opportunity to describe in detail the everyday health promotion practices of their organization, including how participants and target groups were involved in the activities in general. Furthermore, the four case stories were written by the researchers in close dialog with and verified by representatives from the organizations before the analysis began, which helped ensure that the case descriptions accurately reflected the organizations' practices and the participants' perspectives.

**Abbreviations**
NGO: non‐governmental organization
SFS: Swedish Code of Statutes (Svensk författningssamling)
WHO: World Health Organization
WMA: World Medical Association

## 1 Introduction

Health‐promoting settings have been shown to have a positive impact on performance, as well as the individual's health and quality of life [1, 2]. Research also shows variation in how such settings‐based approaches are perceived and implemented in practice, where differences across organizational contexts and target groups are common [3, 4]. Taking a settings‐based approach to health promotion means addressing the contexts within which people live, work, and play [3, 5] and strengthening the capacities of people who exist in different environments [6, 7]. A settings‐based approach encourages connections between people, environments, and behaviors to be explored in everyday practice, for example, workplaces and schools [1, 8, 9]. It highlights relationships between different groups and their interactions, while also facilitating organizational awareness of wider effects on health and sustainability [3, 4]. The approach is further supported by Health‐in‐All Policies frameworks, which emphasize cross‐sectoral collaboration for healthier environments [2]. However, previous research has noted that policy ambitions often outpace empirical knowledge of how such collaborative and settings‐based principles are realized in everyday organizational practice [10, 11]. This highlights the importance of understanding how a settings‐based orientation in practice can increase the likelihood of empowering individuals. This orientation can increase the likelihood of empowering individuals to take care of their health by enabling supportive contextual and organizational conditions that promote health [3, 9, 12]. Parts of this thinking draw from salutogenesis, which considers strengthening resources (factors and processes) within the physical, organizational, and social settings where people exist as central to health promotion efforts, rather than focusing solely on the individuals themselves [13, 14, 15]. Still, research remains limited regarding how such resource‐oriented perspectives are operationalized through concrete organizational practices and interactions.

The respect for individuals as active participants is a core tenet of health promotion processes, characterized by mutually influential relationships between people, their environment, and enabling structures [6, 8]. In relation to this, a systems theory approach helps to understand how internal relations and structures within a system affect one another, clarifying complex organizational dynamics and how this affects individual capacity for action [16]. A holistic, systems‐based perspective is essential for achieving meaningful results and long‐term change in health promotion [10, 17]. Research indicates that systems‐oriented health promotion often struggles to move from conceptual to coherent and sustainable everyday practice [11, 18]. Participation is the key to sustainable change and is considered a crucial resource in addressing the complexity and unpredictability that often characterizes local health promotion initiatives [6]. However, research further shows that participatory ideals are not always matched by meaningful opportunities for influence in practice [19, 20]. It should involve creating conditions that allow everyone affected by an initiative to contribute their experience and engage meaningfully, provided they are willing and able [8, 21]. Empowerment, is here a core strategy that supports individuals or groups to gain influence over their physical, social, and psychological well‐being [12, 22], enabling greater autonomy, personal growth, and active citizenship [6, 7].

Participation and empowerment can also be related to co‐creation in health promotion. Co‐creation represents a multidimensional construct that involves a bottom‐up approach to collaboration, where diversity, mutual trust, openness, autonomy, and respect form the basis for shared expertise, responsibility, and decision‐making [23]. Co‐creation, as well as participation and empowerment, closely align with the theoretical framework of person‐centeredness [24]. Person‐centeredness shares the same humanistic value base and focuses on people in context rather than isolated individuals [10, 25]. The approach is characterized by shared decision‐making, individualized support, and emphasizes organizational readiness to embed person‐centered practices in policy and culture [24, 26, 27]. Despite strong theoretical and normative support, research points out persistent differences between person‐centered intentions and how these principles are implemented in everyday organizational settings [20, 28]. According to Heggdal et al. [29], empowerment in person‐centered interventions is dependent on relational processes, recognition of participants' resources, and opportunities for meaningful involvement in everyday practice. Creating person‐centered systems, therefore requires clear strategies and supportive structures at the health system level [28].

From a systems perspective, person‐centeredness involves not only the interaction between professionals and participants, but also the broader physical, social, and organizational environment that shapes such interactions [24, 30]. Prerequisites for person‐centeredness include an organizational culture grounded in empowerment and continuous development, enabling responsiveness to individuals' previous experiences, needs, and expectations [26, 27]. However, few empirical studies examine how these organizational and environmental conditions interact with relational practices in health‐promoting settings outside traditional healthcare contexts.

Overall, there is still limited research that focuses on how organizations can actively implement health promotion in everyday practice and how this contributes to sustainable health for individuals [11, 18]. Although principles of person‐centeredness, participation, and empowerment are frequently emphasized, there remains a gap in understanding how these concepts are implemented through concrete practices, interactions, and organizational conditions in everyday health promotion settings. Highlighting how the central concepts of participation, empowerment, and person‐centeredness work in different practical contexts can therefore contribute to new insights into how a more equitable and sustainable health promotion practice can be developed. Another contribution is a deeper knowledge for researchers, decision‐makers, and health professionals on how to create and promote more effective and inclusive health promotion strategies at different levels of settings. The aim of the study was therefore to explore different everyday health promotion settings having a specific focus on participation, empowerment, and person‐centeredness in practice.

## 2 Methods

### 2.1 Study Design and Selection

An exploratory qualitative collective case study [31] was conducted with support from case stories as a part of the Storytelling dialog method [32]. The study design was chosen due to the exploratory aim of the study [31], together with the focus on the everyday health promotion practice with a setting approach [11]. The collective case study was characterized by multiple cases being studied simultaneously, with flexibility and adaptation to the cases regarding choice and implementation of qualitative data collection methods [31]. The adaptation to the cases resulted in the use of participating observations and semi‐structured interviews. Participating observations were initially planned for the whole study, but due to the rigorous organizational restrictions related to the second phase of the Covid‐19 pandemic, adjustments had to be made. Digitally semi‐structured interviews with organizational representatives were therefore used as a complement to researchers' previously shorter visits, when participating observations were not an option according to the organizational restrictions for the cases.

This study employed a qualitative multi‐method approach, using multiple qualitative data collection methods to gain a deeper understanding of the phenomenon under study [31]. Methodological considerations were taken to ensure that comparisons between cases were appropriate, despite the use of different qualitative data collection methods [31]. The Storytelling dialog method is useful for knowledge development within the field of health promotion [32] and makes it possible to organize and analyze the qualitative case data gathered from the different data collection methods in a structured way. The Storytelling dialog method includes the use of case stories, which are a recommended tool in combination with other data collection methods in case studies [32].

A strategic but also convenient selection was applied to guarantee the important accessibility to cases of relevance for the aim of the study [31]. The aim of studying health‐promoting settings without delimitation to specific target groups or specific organizations motivated a selection of varying organizations, which aimed at different target groups. Crowe et al. [31] emphasize the importance of a well‐functioning research cooperation and researchers knowing the studied case sites well, which resulted in organizations conveniently but also strategically being invited due to an already established contact with members of the research team. The strategic and convenient selection resulted in four organizations being invited to participate in the study, based on their varying purposes and focus on different target groups (see details in Table 1 below).

**Table 1:** Information about the cases and the data collection.

Setting	Target group	Study participants	Data collection method	Number of researchers involved
A preschool setting	Children 0–5 years	Operations manager together with the coordinator for the health profile of the preschool	Semi‐structured interview	3
A sports education in an upper secondary school setting	Adolescents 13–16 years	Operations manager together with one of the sports instructors	Semi‐structured interview	2
A daily activity setting	Adults with varying disabilities	Operations manager and two mentors working in the organization	Participating observations	2
A workshop series focusing on community development	Senior citizens aged 65+	Varying stakeholders like representatives from the municipality and other senior organizations, senior municipality citizens	Participating observations	2

The study was carried out in accordance with the ethical guidelines of the Swedish Ethical Review Authority and thus the act concerning the ethical review of research involving humans [33]. The Swedish law of research ethics emanates from the WMA Declaration of Helsinki. No formal ethical approval was applied for due to the study's focus on the everyday practice of the participating organizations. Ethical approval is needed if sensitive personal information about participants is collected through research or if the methods used are affecting participants physically or psychologically [33]. The study did not meet any of these criteria.

### 2.2 Presentation of Cases

Case 1: *A preschool setting*. The preschool was located in a municipality with approximately 10,000 inhabitants in the south of Sweden. The preschool was one of nine preschools in the municipality, the only one with a health profile, and the children were divided into three learning units within the organization. The preschool had been built and was organized with support from a holistic health perspective, which had been developed over a 12‐month period prior to the building phase for the facilities. The health concept was systematically developed through a participatory process that included children and parents, municipality inhabitants in general, researchers and university students, as well as preschool staff and management, coordinated and led by the municipality's public health strategist. The preschool had been operating since 2019, was led by a principal, and everyday practice was managed by preschool teachers. A health educator was also employed and supported the preschool teachers regarding the health perspective of everyday practice within the organization.

Case 2: *A sports education in an upper secondary school setting*. The sports education was located in one of two upper secondary schools in a municipality with approximately 13,000 inhabitants in the south of Sweden. The school offered university preparatory programs, vocational training programs, and apprenticeships for pupils aged 16–19. All programs were organized with block reading, which created opportunities for all pupils to participate in a school profile through an individual choice subject. The sports education was a profile through which pupils could incorporate either football, ice hockey, or swimming into their upper secondary program. Approximately 60 pupils participated in the sports education. The everyday practice of sports education was managed by four part‐time employed sports‐specific coaches, who were included in the same work group led by a full‐time employed head coach. The organization also consisted of a management team with expert knowledge and extensive experience in sports medicine from various sports contexts. The management team had developed a holistic health perspective, supported by their own experiences, as well as health‐ and sports‐related research. This perspective had been implemented through collaboration between their company, the school, and a network of collaborative partners in various sports clubs since 2018.

Case 3: *A daily activity setting*. The daily activity setting was located in a municipality with approximately 16,000 inhabitants in the south of Sweden and had a focus on participants with varying neuropsychiatric functionality disabilities since 2020. The organization had one manager and two employed mentors working with approximately 8–10 participants. The organization was characterized by a holistic health perspective, which had been slowly developed through a participatory empowerment process involving participants in a similar daily activity setting, previously led by the same manager in another municipality. The everyday practice was strongly based on activities involving farming, animals, and nature, supported by the manager and two employed mentors in a peer‐tutoring approach. The health perspective had also been communicated with a public health researcher and students during the development process.

Case 4: *Community development for senior municipality citizens*. The workshop series was conducted through three 3‐h thematic workshops during autumn 2020 in a municipality with approximately 85,000 inhabitants in the south of Sweden. The themes emanated from a holistic perspective regarding healthy aging and an elderly‐friendly city, including safe accommodation, meeting places, and general societal accessibility. The municipality was responsible for organizing the workshops and invited various stakeholders, including municipality representatives from different departments, senior citizens from the municipality, representatives from other organizations focusing on the elderly (e.g., NGOs), and researchers. Participants could attend either digitally or in person, with 30–40 participants at each workshop. The aim was to develop an action plan for active and healthy aging in the municipality, based on the outcomes of the workshops.

### 2.3 Research Process

The participating organizations were informed about the study both orally and through written information. The information included a general description of the study, which included the aim of the study, information regarding voluntary participation, confidentiality aspects, and dissemination plans for the findings and contact information to the research leader (Å.B). Informed voluntary consent was thus obtained prior to the organizations and interviewees participating in the study. The data collection was conducted during autumn 2020 and spring 2021. Five of the six researchers were involved in data collection, distributed between the studied organizations as presented in Table 1. One of the researchers interacted somewhat in the activities during the visit to the organizations, while the other researcher oversaw documentation and took observational notes. The observations were conducted with support from a mind map, which was inspired by the description of health promotion with a setting approach [34, 35], a conceptual framework of person‐centered care [36], and the description of a person‐centered culture [37]. The observer took notes related to the content of the mind map continuously and asked complementary questions when needed to understand various situations and everyday activities being observed. The time allocated for observations varied but was approximately 3 × 3 h per organization, depending on the type and accessibility of the organization being observed.

When researchers conducted short visits, these were complemented by a digitally semi‐structured interview. The participants were asked to describe the organization in detail, with focus on, for instance, everyday practice, the purpose and history of the organization, as well as challenges, success factors for goal achievement, and opportunities for organizational development. The organizational representatives were also asked to share their thoughts about key concepts of health promotion and person‐centeredness, emanating from the mind map that was used for the observations, and relate the concepts to the described everyday practice of the organization. One of the researchers was in charge of the interview, and one of the others was assisting and taking complementary notes. The assisting researcher also had the opportunity to ask complementary questions when needed. The interviews started with a wide and open start‐up question, followed by a selection of follow‐up questions used in different ways depending on the initial description coming from the interviewees, in order to gain a deeper description and understanding. The interviews were recorded digitally, after the participants consented to be recorded and the interviews lasted approximately 90 min.

The collected data (audio recordings and notes) were used as support for writing case stories, inspired by the guidelines for the Storytelling dialog method [32]. The case stories were written by researchers and presented to and verified by participating organizational representatives before starting the analysis.

### 2.4 Analysis

The case stories constituted the unit of analysis and were analyzed through a customized version of the structured analysis process of the Storytelling dialog method [32]. The fifth step (Insights cards) and seventh step (Plenary) were not useful since only one group of researchers took part in the process. All six researchers participated in the storytelling dialog, but one researcher (K.N.) documented the dialog as a story recorder and acted as support for a critical perspective throughout the interpretation process of the analysis. The story recorder had no previously established contact with the selected cases and was not involved in the data collection or writing case stories.

The storytelling dialog started with the four case stories being orally presented to the research group in total. The presentation was then followed by a reflection circle where the researchers were given the opportunity to orally reflect on the content of the different case stories on a more general level. The structured dialog was then conducted with support from the questions: *What, Why*, and *So what?* The analysis was characterized by an inductive approach and the reflections in the research group resulted in two complementary themes.

## 3 Findings

The analysis of the case studies resulted in the findings being divided into two themes representing complementary perspectives of health promotion with a setting approach. The first theme included interconnected structural, organizational, and relational aspects of the everyday practice in the organizations and was, therefore, named *Practice‐embedded prerequisites for a setting approach*. The second theme was named *An experiential and behavioral process supporting health development in settings*, thus complementing the first with a process‐oriented description of health development for people in the target groups of the studied organizations. All in all, the findings show how practice‐embedded prerequisites for a setting approach enable people's participation, which gives rise to their internalized psychosocial experiences. Experiences that foster the participants' active engagement and sustained participation in the organizations' everyday activities and thereby create conditions for health development through an experiential and behavioral process in settings over time.

### 3.1 Practice‐Embedded Prerequisites for a Setting Approach

The identified practice‐embedded prerequisites for health promotion with a setting approach were organized in the four dimensions: *Society, Organization, Staff*, and *Interaction between staff and participants*.

*The society* was described as a facilitating support for health promotion on organizational level. It was, for instance, exemplified by municipalities aiming for marketing their local context with a health perspective. Different kinds of cooperation with other organizations were also highlighted as important support for health‐promoting organizations. Sometimes the cooperation was related to different levels of the administrative hierarchy within the municipality organization, and at other occasions, cooperation with other organizations was described as important.

*The organization* shaped various contextual prerequisites for health promotion. Related to a leadership being characterized by a person‐centered approach, that facilitated the staff's opportunities to carry through the everyday practice of the organization. Allocated time and a long‐term process distinguishing the everyday practice interaction between staff and participants was also described as a prerequisite on the organizational level. The organization also needed to be characterized by clarity regarding aims and goals. The physical environment, both indoor and outdoor, of the organizations was also emphasized as important for health promotion opportunities and activities. The environment needed to be supportive, which meant appropriate for and adapted to the daily practice of the organizations. The location and accessibility of the organizations were also identified as important aspects. Prerequisites for health promotion on the organizational level were also related to having a health perspective, which permeated the organizations in various ways. The case stories showed that all participating organizations had support of public health competence in the implementation process of the health promotion profile, or as a reflective dialog partner, further into the implementation process of the organizations.

*The staff* were important as facilitators for health‐promoting processes for the participants, through their responsibility for the everyday practice of the organizations. Their competence, engagement, as well as ability to be flexible and adapt everyday practice to the needs of the participants were considered highly relevant for health promotion action. The staff's personal values also needed to be in coherence with the values of the health‐promoting character of the organization, to be authentic. Multi‐professionalism was considered important, and accessibility to health promotion expertise was supported, for instance, by the pedagogical staff in the educational settings.

*The Interaction between staff and participants* was considered the core of health‐promoting processes. This interaction needed to be characterized by supportive relationships and a continuous dialog with mutual reflection. The dialog needed to include communication regarding activities and tasks based on the participants' personal needs and abilities. The case stories showed the importance of “putting the participants in the center” and “meeting the needs of the individuals.” Low‐affective treatment was also used for individual adaptation and promotion of the individuals' feelings that they succeed in the things they do. Voluntary participation in various activities was also described as a means for meeting the participants' specific needs.

### 3.2 An Experiential and Behavioral Process Supporting Health Development in Settings

The description of the practice‐embedded prerequisites for a settings approach could be related to health‐promoting individual and group experiences. The organizational stories show how the identified factors of relevance, on different levels, create opportunities for health‐promoting processes for the participants of the different organizations. These factors can thus be considered characteristics of supportive environments that create opportunities for empowerment, participation, and person‐centeredness in processes for health promotion in practice. The characteristics of the supportive environments were related to the participants' experience of *being included*, a sense of *belongingness* and *motivational characteristics*, which therefore were considered beneficial in health‐promoting processes for the participants of the organizations.

*Being included* illustrated the participants' experience of the organization being characterized by openness, the experience of adaptation to the group, as well as the individual participants, and the experience of being seen and heard. *Belongingness*, on the other hand, is related to the participant's experience of community, trust, and security. Participants' experience of *motivation and meaningfulness* was also described as important, based on these aspects being the driving force for the participants' commitment to the organization. The importance of participants experiencing meaningful activities and having a sense of pride over their accomplishments was emphasized.

The description of the participants' experience of *being included*, a sense of *belongingness*, and *motivational characteristics* in the settings was related to individuals and target groups acting in the organizations in general and specifically in relation to their health development.

Participants' experience of being included, a sense of belongingness and motivation were thus described as foundations for the participants acting in the organization, as well as their opportunities for health development over time. The participants' daring and wanting to engage through *active participation* in general and particularly regarding *raising their voice* for exercising influence together with *standing up for their own needs*, were considered important for empowerment and opportunity for health development. Opportunities for *health development* were also considered a long‐term goal and included willingness and daring to change and develop through a continuous *lifelong learning* process, *self‐realization, thrive*, and *flourish*. Development at the participants' own pace was, however, considered important for their growth and ability to take individual responsibility for various issues.

## 4 Discussion

The findings show how different prerequisites, from basic societal and organizational conditions to meaningful personal efforts and interactions, create the basis for health‐promoting processes in settings. These prerequisites correspond to what was conceptualized in the findings as *practice‐embedded prerequisites for a setting approach*, embedded in societal, organizational, staff‐related, and relational dimensions of everyday practice. The findings can be understood with support from the process‐oriented vertical logic of the Log Frame model (Green, Cross, Woodall & Tones, 2019). The findings show how experiential foundations, such as a sense of inclusion and motivation, lay the groundwork for increased participant engagement and sustainable health development. When supportive settings promote empowerment, participation, and person‐centeredness, the person can move from feeling a sense of belonging to taking an active role in their health development. In line with the second theme identified in the findings, this movement reflects *an experiential and behavioral process supporting health development in settings*.

Across the studied settings, this process can be understood as co‐creation [23], when participants, staff, and organizational actors jointly shape health‐promoting activities through dialog, shared decision‐making, and iterative adaptation over time. Co‐creation can thus be seen as the dynamic link between practice‐embedded prerequisites and the experiential and behavioral processes emerging within the setting. Research has shown that co‐creation and health promotion are interrelated and mutually reinforcing approaches, providing a foundation for participatory and collaborative practices that are sensitive to context and grounded in knowledge‐based practice, thereby addressing the inherent complexity of health [38]. This progression is supported by Downey et al. (2021), who show that person‐centered interventions focusing on personal relationships and sense‐making contribute to improved well‐being and sustained engagement. This creates the conditions for new, improved, and sustainable health‐promoting efforts to achieve lasting, positive health effects for all members of a target group over time [23]. The analysis of the four case stories from different everyday health promotion settings shows that *participation, empowerment*, and *person‐centeredness* are closely intertwined in practical health promotion work. The concepts are found in the various dimensions of prerequisites, from societal structures to individual meetings, and together constitute a dynamic foundation for sustainable health promotion. These dimensions reflect how practice‐embedded prerequisites operate across levels, shaping the contextual conditions within which experiential and behavioral processes evolve. It is in the mutual interaction between structural conditions, organizational culture, and personal values and experiences that supportive environments can emerge. The findings are supported by previous research that emphasizes the importance of a holistic approach and intersectoral collaboration in health promotion efforts [1, 2]. This is further underlined by Bloch et al. [4], who argue for the “super‐setting” approach, where coordinated actions across multiple settings create synergistic health effects that promote sustainability. By promoting environments where the individual's voice is given space, where relationships and co‐creation between participants and staff are genuine, and where structures enable development, the conditions for real change are created, not only at the individual level, but also for public health in society [16, 30].

The findings also highlight the importance of experiential foundations in strengthening participants' opportunities for increased health and agency. A sense of inclusion, belonging, and motivation emerges as key factors in creating supportive environments where participants feel seen and heard [6, 8]. These experiential dimensions correspond to the core elements of the identified experiential and behavioral process, where psychosocial experiences foster active engagement in the setting. The findings are in line with person‐centered principles, as they are based on the individual's unique needs and circumstances, which are taken into account through shared decision‐making [24, 30]. Also, Downey et al. (2021) demonstrate how meaningful social engagement within a structured program can foster a sense of connection and self‐worth, reinforcing the value of highlighting these experiential dimensions in empowerment processes. When participants experience being included and motivated, the likelihood increases that they will engage in change processes, develop their skills, and take greater responsibility. Through this behavioral engagement within supportive settings, health development becomes an ongoing and contextually embedded process rather than a linear outcome [14, 27]. This creates conditions for achieving more comprehensive and sustainable health improvements, not only at the individual level but also in the larger group or organization through health‐promoting settings [1]. Deeper forms of empowerment and participation are reflected when participants express their needs and preferences and pursue their own and the group's concerns [12]. This connection is also supported by Avery, Sjögren Forss, and Rämgård [6], who show that empowering participants through co‐creation in local health promotion labs leads to increased agency and ownership.

*Participation* is described in our findings both as a prerequisite for and as a result of health‐promoting settings. This dual role reflects the interplay between practice‐embedded prerequisites and experiential and behavioral processes for health development. In dialog‐based and respectful interactions, where participants are allowed to influence the content and design of interventions (shared decision‐making), participation is a given way of working. Such reflecting co‐creative processes are knowledge, experiences, and responsibilities shared between participants and professionals [8, 9]. At the same time, participation is not something static; it is not certain that active participation occurs simply because the opportunity has been offered [19]. Rather, participation develops as participants feel listened to and are given the opportunity to contribute to a context or on an issue that concerns them [6, 20]. In this way, participation also becomes a way to strengthen the empowerment of individuals or groups, since it is through active participation and shared decision‐making that the person or group is allowed to influence, develop, feel a sense of belonging, and get involved [12, 22]. Bloch et al. [4] support this understanding by emphasizing that participation across multiple settings is key to creating comprehensive and engaging health promotion interventions.

In health promotion, *empowerment* is a promoting process, not a condition or a goal in itself, but something that occurs based on the approach that health promotion is grounded on [5]. Our findings show that different contexts, settings, and target groups can promote empowerment in various ways, through the described experiential and behavioral engagement and practice‐embedded prerequisites of different levels. At the societal level, empowerment requires overall support, for example, from the municipality, which creates structural conditions for individuals and organizations to work on health promotion in a societal context. At the organizational level, collective empowerment means creating space for staff to work together in the long‐term and a relationship‐oriented manner, which in turn enables both staff and participants to gradually take greater responsibility for co‐creation and influence their own work and life situations. It is about building trust, creating safe environments, and promoting people's capacity to act based on their resources and goals [12, 22]. Individual empowerment is expressed here in everyday life, when the participant, through supportive relationships, dares to take a place, express their needs, and participate in decisions that affect their own lives [29]. This progression from passive recipient to active co‐creator is a core aspect of health promotion work and emphasizes how co‐creation emerges through small, but continuous steps of empowerment in everyday practice [21]. This process mirrors the findings of Avery, Sjögren Forss, and Rämgård [6], where participants, through iterative involvement, evolved into proactive contributors in community‐based health initiatives.

Beyond individual empowerment, the findings also point to the importance of collective empowerment, where co‐creative processes enable groups of participants and professionals to jointly influence practices, norms, and organization through new out‐of‐the‐box context‐specific health‐promoting practices addressing co‐defined needs at group level [23]. With support from co‐creation, empowerment can extend beyond individual participation and person‐centered encounters to support structural change within health‐promoting settings. There is a strong emphasis and urgency for action at the global level to support people in taking control of their lives and health, as part of creating sustainable well‐being societies committed to equitable health [39]. There is also an identified need for competence development regarding implementing co‐creation into real‐life processes for health promotion [40]. Darlington et al. [40] have also identified a need for a shared conceptualization of co‐creation and its associated processes that support increased inclusivity, engagement, and mutual sense‐making in an open action‐oriented approach. More research focusing on opportunities and challenges of using co‐creation in health promotion for health equity and sustainable development is, however, needed [38].

Further, the findings show that *person‐centeredness* is a fundamental principle in health‐promoting settings, especially in the encounter between staff and participants. Person‐centeredness constitutes the relational mechanism through which practice‐embedded prerequisites are translated into experiential and behavioral processes. It is emphasized that staff need to see and adapt to the unique needs of the person, which is in line with the definition of person‐centeredness, where the participant is seen as an active actor in their health process [24]. Person‐centeredness is manifested at the organizational level, through a value base and work structure that enables individually adapted interventions, and in practice, through the staff's attitude and flexibility [10, 26, 27]. The physical and social environment plays a significant role in reinforcing the participant's feeling of being seen and confirmed, which can contribute to increased meaningfulness and strengthened commitment [8, 15]. A person‐centered approach is also in line with WHO's emphasis on individually tailored, but also contextually relevant health interventions and strategies for health promotion that are based on respect for the individual's rights, own values and preferences for increased health and well‐being in the person's everyday life [17, 39, 41]. Downey et al. (2021) show how person‐centered programs can foster trust and personal development by focusing on relationship‐building and meaningful engagement. By clarifying how experiential foundations support person‐centered health promotion processes, the findings demonstrate the importance of creating safe conditions for individuals to grow and achieve sustainable change at individual, group, organizational, and societal levels.

### 4.1 Practical Implications

The findings confirm the complexity of health promotion with a setting approach and the need for a whole multi‐level system, as well as a process‐oriented approach when organizing health‐promoting settings in practice. The findings also show how prerequisites for health development on individual and group levels are embedded in the interaction between staff and participants during everyday practice of the organizations, highlighting the need for staff to have relational skills and competence for co‐creation, as well as awareness of their role for participants' opportunities for empowerment and health development. Health‐promoting settings also need, however, person‐centered leadership, as well as complementary health promotion expertise supporting managers and professionals working with co‐creation in the settings on an everyday basis. Organizations therefore need to ensure ongoing professional health promotion support that provides guidance, facilitates learning processes, and strengthens the integration of health promotion principles in daily work with support from a whole system approach. The similarities between the analyzed organizations, despite their differences, show how knowledge development through exchange of experiences can be used for continuous development of context‐specific health‐promoting practice with a setting approach. Different health‐promoting settings, which can contribute to community development through super‐settings for health in the longer term.

### 4.2 Trustworthiness

The study's trustworthiness [42] is strengthened by using an exploratory qualitative case study design, as it was well‐suited to the study's exploratory purpose of describing and understanding everyday health promotion practice within different types of organizations [31]. The selection of participating organizations was both strategic and convenient, which was done to ensure access to relevant cases and to enhance the opportunity to shed light on different aspects of the phenomenon, and this is seen as a strength of the study. However, the selection strategy entailed a certain risk of limited *transferability* of the findings, since there are many more health contexts that are not represented in this study. The selection procedure nevertheless enabled an in‐depth study of the contexts and complex processes that characterize the participating organizations, which is central to understanding the many dimensions of health promotion practice. The clear methodological description of the research process and the four cases increase the *credibility* and *transferability* of the study.

Data collection through observations and interviews enabled adaptation of data collection to each case's unique conditions for participation. The strength of the adapted data collection was its flexibility, which was particularly valuable during the Covid‐19 pandemic when the study was conducted, and restrictions made it difficult to conduct observations within certain organizations. At the same time, the case study design required careful consideration of how data from different cases could be compared and analyzed in a meaningful way, without missing specific contextual details. The two adapted qualitative data collection methods may, thus, have affected data depth and interactional dynamics. In particular, the risk of fragmented narratives and reduced contextual richness was identified. To address this, data were transformed into case‐based stories prior to analysis. This process supported analytical coherence by preserving temporal and contextual relationships within each case. The use of the Storytelling dialog method [32] further enhanced transparency by making interpretive steps explicit. However, limitations remain regarding the absence of in‐site observation of two organizations, which may have constrained the interpretation of non‐verbal cues. However, the representatives were given the opportunity to review and validate their own narratives, but they were not involved in confirming the findings of the subsequent analysis. This may have further limited the study's confirmability. Consequently, it is a limitation that only the researchers themselves participated in the analysis. The organization representatives could have been included, for example, through member‐checking, and there is therefore a risk that certain nuances or alternative interpretations were not captured.

Ethical considerations were made in planning and implementation of the study to ensure informed consent and confidentiality, which strengthens the *credibility* of the study from an ethical perspective. The clear information provided to the participants, including the purpose of the study, the voluntary nature, and how the findings would be used, also strengthens the *credibility* of the study.

## 5 Conclusion

This study conceptualizes health promotion in everyday settings through two interrelated themes: *Practice‐embedded prerequisites for a setting approach* and *An experiential and behavioral process supporting health development in settings*. The findings show how societal support, organizational structures, staff competence, and relational interaction form embedded prerequisites that create supportive environments for participation, empowerment, and person‐centeredness.

The findings indicate that experiences of inclusion, motivation, and a sense of belonging play an important role in supporting continued engagement and perceived agency among participants. Further, the findings highlight possible pathways through which supportive environments may contribute to ongoing engagement, lifelong learning processes, self‐realization, flourishing, and health within everyday settings.

The findings also show that, within the studied cases, environments in which individuals feel heard, respected, and able to contribute meaningfully are associated with a shift from a more passive role toward increased engagement in health‐related processes. Our study points to the relevance of integrated, empowerment‐oriented, and person‐centered approaches in health promotion practice within everyday settings.

Future research should further explore everyday health promotion settings that emphasize participation, empowerment, and person‐centeredness in practice. To deepen the understanding of this phenomenon, studies conducted in different organizational contexts and types of activities would be valuable, as well as more in‐depth analyses and longitudinal designs. Such research could capture the complexity of these processes over time and across settings, thereby contributing to theoretical advancement while also generating insights that may inform practice and contribute to discussions on broader societal implications.

## Author Contributions

**Petra Nilsson Lindström:** conceptualization, investigation, methodology, formal analysis, writing – original draft, project administration, validation, funding acquisition. **Sophie Schön Persson:** conceptualization, investigation, methodology, formal analysis, writing – review and editing. **Johanna Sjöbeck:** conceptualization, investigation, methodology, formal analysis, writing – review and editing. **Matilda Ahl:** conceptualization, investigation, methodology, formal analysis, writing – review and editing. **Kerstin Nilsson:** conceptualization, methodology, formal analysis, writing – review and editing. **Åsa Bringsén:** conceptualization, investigation, methodology, formal analysis, writing – original draft, project administration, validation, funding acquisition.

## Ethics Statement

All participants gave their informed consent to the data collection, in line with the Swedish Law of Research Ethics [33], and ethical approval was not needed for this study.

## Conflicts of Interest

The authors declare no conflicts of interest.

## Data Availability

The data that support the findings of this study are available from the corresponding author upon reasonable request.
